# Feasibility of accelerated T2 mapping for the preoperative assessment of endometrial carcinoma

**DOI:** 10.3389/fonc.2023.1117148

**Published:** 2023-07-26

**Authors:** Zanxia Zhang, Jie Liu, Weijian Wang, Yong Zhang, Feifei Qu, Tom Hilbert, Tobias Kober, Jingliang Cheng, Shujian Li, Jinxia Zhu

**Affiliations:** ^1^Department of Magnetic Resonance Imaging, The First Affiliated Hospital of Zhengzhou University, Zhengzhou, China; ^2^Magnetic Resonance Collaboration, Siemens Healthcare Ltd., Beijing, China; ^3^Advanced Clinical Imaging Technology, Siemens Healthcare AG, Lausanne, Switzerland; ^4^Signal Processing Lab 5 (LTS5), Ecole Polytechnique Fédérale de Lausanne, Lausanne, Switzerland; ^5^Department of Radiology, Lausanne University Hospital and University of Lausanne, Lausanne, Switzerland

**Keywords:** endometrial cancer, T2 mapping, magnetic resonance imaging, diffusion weighted, apparent diffusion coefficient

## Abstract

**Objective:**

The application value of T2 mapping in evaluating endometrial carcinoma (EMC) features remains unclear. The aim of the study was to determine the quantitative T2 values in EMC using a novel accelerated T2 mapping, and evaluate them for detection, classification,and grading of EMC.

**Materials and methods:**

Fifty-six patients with pathologically confirmed EMC and 17 healthy volunteers were prospectively enrolled in this study. All participants underwent pelvic magnetic resonance imaging, including DWI and accelerated T2 mapping, before treatment. The T2 and apparent diffusion coefficient (ADC) values of different pathologic EMC features were extracted and compared. Receiver operating characteristic (ROC) curve analysis was performed to analyze the diagnostic efficacy of the T2 and ADC values in distinguishing different pathological features of EMC.

**Results:**

The T2 values and ADC values were significantly lower in EMC than in normal endometrium (bothl *p* < 0.05). The T2 and ADC values were significantly different between endometrioid adenocarcinoma (EA) and non-EA (both p < 0.05) and EMC tumor grades (all p < 0.05) but not for EMC clinical types (both p > 0.05) and depth of myometrial invasion (both p > 0.05). The area under the ROC curve (AUC) was higher for T2 values than for ADC values in predicting grade 3 EA (0.939 vs. 0.764, p = 0.048). When combined T2 and ADC values, the AUC for predicting grade 3 EA showed a significant increase to 0.947 (p = 0.03) compared with those of ADC values. The T2 and ADC values were negatively correlated with the tumor grades (r = -0.706 and r = -0.537, respectively).

**Conclusion:**

Quantitative T2 values demonstrate potential suitability in discriminating between EMC and normal endometrium, EA and non-EA, grade 3 EA and grade 1/2 EA. Combining T2 and ADC values performs better in predicting the histological grades of EA in comparison with ADC values alone.

## 1 Introduction

Endometrial carcinoma (EMC) is a common malignant tumor of the female reproductive system and has continued increasing incidence and mortality rates worldwide (1). The treatment strategies and survival outcomes of patients with EMC are associated with the histologic and pathologic grades of EMC, the status of myometrial invasion depths, cervical stromal invasion, and lymph node metastases (2). Magnetic resonance imaging (MRI) is a common and standard imaging modality for EMC’s diagnosis and preoperative staging (3). Diagnoses based on conventional MRI rely on comparing signal intensities from different tissues, which can be easily affected by equipment variability and scan parameters. Quantitative MRI methods, such as diffusion-tensor imaging, dynamic contrast-enhanced analysis, and amide proton transfer-weighted MRI, have been used for clinical diagnoses, tumor progression, and treatment responses of patients with EMC (4–7).

T2 mapping is a quantitative MRI method that measures the transverse relaxation time (T2) of various tissues, which is displayed as image voxels on parametric maps (8). The T2 or transverse relaxation time is related to the lifetime of the magnetization component in the plane perpendicular to the magnetic field direction, which is zero at equilibrium. Conventional T2 mapping is performed with a multiplanar multiecho spin echo sequence, which requires long scan times to acquire the entire k-space. GRAPPATINI is an accelerated T2 mapping method that significantly shortens T2 mapping times by combining the generalized auto-calibrating partially parallel acquisition (GRAPPA) technique with the model-based accelerated relaxometry by iterative nonlinear inversion (MARTINI) technique using k-space undersampling (9). The efficacy of the accelerated T2 mapping technique has been reported for the prostate, cervix, pancreas, rectum, and knee (8, 10–12). E.g., Mai et al. assessed quantitative T2 values derived from accelerated T2 mapping to diagnose and grade prostate cancer. Diverse T2 values were observed between different grades of prostate cancer and suggested that quantitative T2 values may help discriminate malignant lesions from other benign pathologies (10). Moreover, Li and colleagues used accelerated T2 mapping and diffusion measures for evaluating cervical cancer (CC) classification, grade and lymphovascular space invasion (LVSI), finding lower quantitative T2 values in poorly differentiated CC and LVSI-positive CC (8). However, the clinical value of this technique in EMC remains unclear.

This study aimed to determine the quantitative T2 values for endometrial cancer and normal endometrium using a novel accelerated T2 mapping, and their evaluation with regard to detection, classification, and grading of endometrial cancer in comparison to the corresponding apparent diffusion coefficient (ADC) value.

## 2 Materials and methods

### 2.1 Study participants

This prospective study was approved by the institutional review board of our hospital. Written informed consent was obtained from all study participants. This study included 109 patients who were diagnosed with EMC between December 2019 and October 2021 at our hospital. The inclusion criteria were as follows: (1) patients without dilation and curettage, surgery, or other related treatments before the MRI examination; (2) patients suspected of having EMC; (3) patients with data from all MRI sequences relevant to this study; (4) patients that underwent surgical treatment within two weeks after the MRI examination; and (5) patients with a confirmation of EMC based on the histopathologic examination after surgery. The exclusion criteria were as follows: (1) patients with small lesions (n = 15), (2) poor image quality that did not meet the requirements for analysis (n = 12), (3) histologic subtype or pathologic grading that could not be determined by the histopathologic examination (n = 15), and (4) patients treated with radiotherapy and chemotherapy before the MRI examination (n = 11). Finally, 56 patients (mean age: 55 ± 7 years; range: 27–64 years) were enrolled in the study. Among these, 35.7% (20/56) were premenopausal women, and 64.3% (36/56) were postmenopausal women. The mean time interval between MRI acquisitions and surgery was 7.5 days (range: 2–14 days). The study also included 17 age-matched healthy volunteers during the same period, and none of the volunteers showed any abnormalities in the uterus or adnexa according to the ultrasound or MRI examinations. The mean age of the volunteers was 53 ± 10 years (range: 27–64 years). Among the 17 healthy volunteers, 41.2% (7/17) were premenopausal women, and 58.8% (10/17) were postmenopausal women.

### 2.2 Image acquisition

The pelvic MRI examinations were performed using a 3T MRI scanner (MAGNETOM Skyra, Erlangen, Germany) equipped with an 18-channel phased-array body coil. The patients were scanned in a head-first supine position. All patients of childbearing age and healthy volunteers underwent MRI examinations between days 5 and 14 after the end of menses, whereas the timing of the MRI examination was not restricted for the menopausal patients and the healthy volunteers (13). The patients were instructed to fast for four to six hours before the MRI acquisition. The MRI scans were performed bilaterally from the superior margin of the iliac wing to the level of the femoral neck by using the conventional T2-weighted turbo spin echo sequence, DWI, and a prototypic T2 mapping sequence. In three diffusion directions, DWI was acquired using an echo planar sequence with b-values of 50 and 1000 s/mm^2^. T2 mapping was performed using the prototypic multiecho sequence accelerated 10-fold with the GRAPPATINI technique by using a parallel imaging factor of 2 and an undersampling factor of 5. The apparent diffusion coefficient (ADC) and T2 parametric maps were automatically generated inline after data acquisition. The MRI sequence parameters are shown in **Table 1**.

**Table 1:** Magnetic resonance imaging acquisition parameters.

Parameters	Conventional T2W TSE imaging	Diffusion-weighted EPI	GRAPPATINI-accelerated T2 mapping
TR (ms)	3000	3910	4680
TE (ms)	116	57	10, 20, 30, 40, 50, 60, 70, 80, 90, 100, 110, 120, 130, 140, 150, 160
Field of view (mm2)	180×180	320×240	220×220
Slice thickness (mm)	4	4	4
Matrix	384×288	192×144	320×240
Flip angle (°)	160	180	180
Bandwidth (Hz/pixel)	200	1002	195
Acquisition time	3 min 20 s	1 min 58 s	4 min 17 s

T2W, T2-weighted; TSE, turbo spin-echo; EPI, echo-planar imaging; TR, repetition time; TE, echo time; GRAPPATINI, combined generalized autocalibrating partially parallel acquisition and model-based acceleration by iterative nonlinear inversion.

### 2.3 Image analysis and measurements

The data analysis of all MRI images was performed using *syngo*.via software (Erlangen, Germany). Two radiologists with eight and five years of experience in pelvic MRI independently performed the quantitative analysis by using the double-blinded method. Statistical analysis was performed using the average values of the measurements by the two radiologists. The EMC lesions, junctional zone (JZ), and outer myometrium (OMM) were identified on the ADC and the T2 maps on the basis of the T2-weighted imaging (T2WI) and DWI scans, respectively. The regions of interest (ROIs) were manually drawn for the EMC lesions, JZ, and OMM of all patients with EMC, and each ROI included more than 25 pixels. Furthermore, the shape and position of the ROIs on the ADC and the T2 maps were consistent. The ROIs for the EMC lesions were drawn along the largest cross-section of the lesions. In the case of thin or unclear JZ, the ROIs were drawn around the muscle tissue adjacent to the EMC lesion (medial 50% of the muscle layer) (14). The cystic, necrotic, and hemorrhagic areas were avoided as much as possible while drawing the ROIs. Normal endometrial ROIs were drawn for healthy individuals by using the T2WI scans as references. An elliptical ROI was drawn on the ADC and T2 maps along the largest section where the endometrium was clearly visible.

### 2.4 Pathologic evaluation

All 56 patients with EMC underwent standard surgery consistent with the current FIGO staging criteria (15). The histopathology of all the surgical specimens was evaluated by a pathologist with >10 years of experience, who was blinded to the results from the MRI diagnosis. The histopathologic subtypes, pathologic grading, and myometrial invasion depth of the surgical specimens were used as the reference standards.

### 2.5 Statistical analysis

SPSS 21.0 (IBM Corp., Armonk, NY, USA) and MedCalc (MedCalc Software, Mariakerke, Belgium) were used for the statistical analyses. The Shapiro–Wilk normality test was used to determine normal or skewed data distribution for each group. The nonnormally distributed data were compared using the Kruskal–Wallis *H* test and the Mann–Whitney *U* test, and the Bonferroni correction method was used to obtain the adjusted *p* values. Receiver operating characteristic (ROC) curve analysis was performed to analyze the diagnostic efficacy of the T2 and ADC values. Each parameter’s discriminating power was quantified using the area under the ROC curve (AUC). The DeLong test was performed to compare the differences in the diagnostic efficacy of each parameter. Pearson’s correlation coefficient was used to evaluate the correlation between the T2 and ADC values. Spearman correlation was applied to analyze the association between the T2 and ADC values with different histological grades. The intraclass correlation coefficient (ICC) was used to evaluate the agreement in the quantitative measurements between the two radiologists, in which ICC ≥ 0.75 was considered a good agreement. Also, a *p* value < 0.05 was considered statistically significant.

## 3 Results

### 3.1 Patient characteristics

This study enrolled 56 patients with a postoperative pathologic diagnosis of EMC and 17 healthy volunteers. The mean ages of the patients with EMC (55 ± 7 years) and healthy volunteers (53 ± 10 years) were not significantly statistically different (t = 0.821; *p* = 0.421). The clinical characteristics of the patients are shown in **Table 2**.

**Table 2:** Clinical characteristics of the 56 enrolled patients with endometrial carcinoma.

Characteristics	Number of patients
Pathology	
Endometrioid adenocarcinoma	46
Mucinous adenocarcinoma	2
Serous adenocarcinoma	3
Clear cell carcinoma	1
Carcinosarcoma	2
Undifferentiated carcinoma	2
Clinical type	
Type I	36
Type II	20
Histologic grade	
Well-differentiated (G1)	19
Moderately differentiated(G2)	17
Poorly differentiated(G3)	10
Myometrial invasion	
Superficial (<50%)	40
Deep (≥50%)	16
FIGO stage	
IA	27
IB	12
II	9
IIIA	3
IIIC1	4
IV	1

FIGO, International Federation of Gynecology and Obstetrics.

### 3.2 T2 values and ADC values of the uterine wall, normal endometrium, and EMC

**Figure 1:**
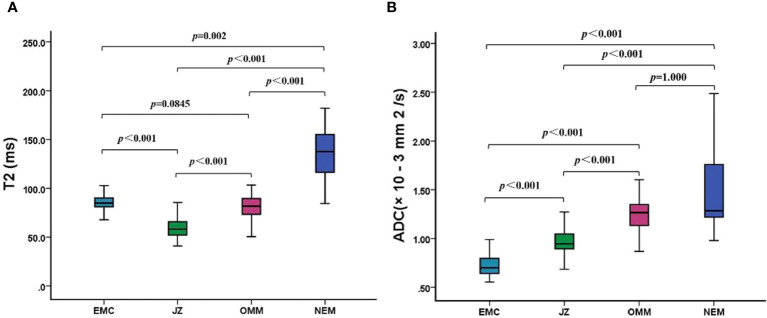
T2 and ADC values of EMC, JZ, OMM, and NEM. **(A)** Boxplots show the T2 values of EMC, JZ, OMM, and NEM. **(B)** Boxplots show the ADC values of EMC, JZ, OMM, and NEM. ADC, apparent diffusion coefficient; EMC, endometrial carcinoma; JZ, junctional zone; OMM, outer myometrium; NEM, normal endometrium.

**Figure 2:**
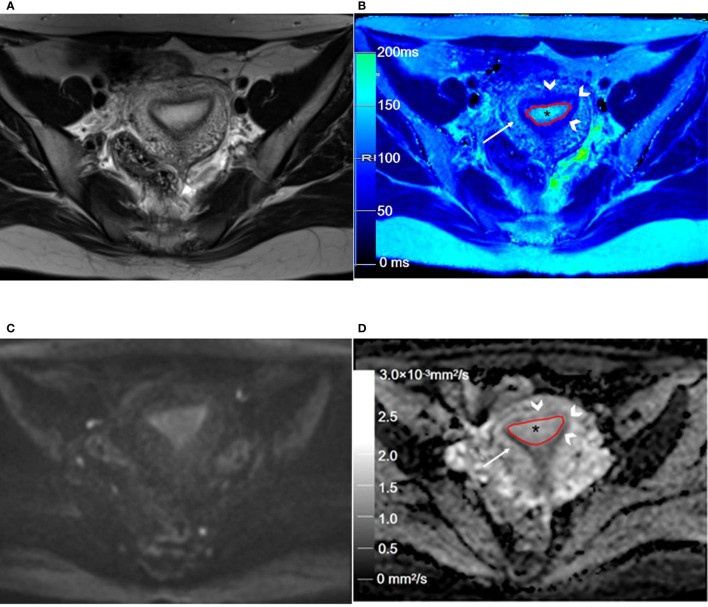
Representative T2WI and DWI and the corresponding axial T2 and ADC maps of the endometrium region in a 46-year-old healthy volunteer. **(A)** Representative axial T2-weighted image. **(B)** The axial T2 map shows the normal endometrium (outlined in red, * in **B**), and the outer myometrium hyperintense (arrow in **B**) is hyperintense. The hypointense JZ (arrowheads in **B**) is continuous and intact. **(C)** Axial DWI (b = 1000 s/mm^2^). **(D)** The axial ADC map shows the normal endometrium (outlined in red, * in **D**), and the outer myometrium hyperintense (arrow in **D**) is slightly hyperintense. The hypointense JZ (arrowheads in **D**) is intact. T2WI, T2-weighted imaging; DWI, diffusion-weighted imaging; ADC, apparent diffusion coefficient; JZ, junctional zone.

The T2 and ADC values of the uterine wall, normal endometrium, and EMC are shown in **Table 3** and **Figure 1**. The T2 values of the EMC were significantly lower than those for the normal endometrium (*p* = 0.002) and significantly higher than those for the JZ (*p* < 0.001); however, significant differences were not observed between the T2 values of the EMC and the OMM (*p* = 0.845) (**Figure 1A**). Also, the T2 values of the JZ were significantly lower than those of the EMC, OMM, and normal endometrium (all *p* < 0.001) (**Figure 2**). The ADC values of the EMC were significantly lower than the ADC values of the uterine wall layers and normal endometrium (all *p* < 0.001) (**Figure 1B**). In addition, the ADC values of the JZ were significantly lower than those of the normal endometrium and OMM (both *p* < 0.001). Further, the ICC analysis showed a good agreement between the two radiologists for the T2 and ADC values of the EMC, normal endometrium, JZ, and OMM (**Table 3**).

**Table 3:** T2 and ADC values for NEM, JZ, OMM, and EMC.

Tissue	T2 (ms)	ADC (×10-3mm2/s)	Intraclass Correlation Coefficient (ICC, 95% confidence interval)	
			T2 value	ADC
NEM	152.0 ± 58.0a	1.52 ± 0.50a	0.881 (0.759,0.943)	0.851 (0.703,0.928)
JZ	58.6 ± 9.9a	0.97 ± 0.13a	0.934 (0.709,0.986)	0.942 (0.739,0.988)
OMM	81.8 ± 12.4	1.24 ± 0.19a	0.798 (0.610,0.901)	0.793 (0.600,0.898)
EMC	86.8 ± 10.4	0.73 ± 0.13	0.854 (0.709,0.930)	0.806 (0.623,0.905)

a Significantly different from the corresponding value of EMC.

T2, transverse relaxation time; ADC, apparent diffusion coefficient; EMC, endometrial carcinoma; JZ, junctional zone; OMM, outer myometrium; NEM, normal endometrium.

### 3.3 Comparison of T2 values and ADC values of the different pathologic features

**Figure 3:**
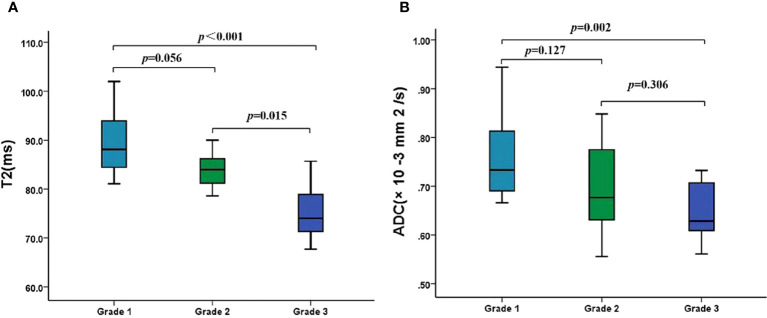
The T2 and ADC values of different histologic grades of EA. **(A)** Boxplots show the T2 values of different histologic grades of EA. **(B)** Boxplots show the ADC values of different histologic grades of EA. ADC, apparent diffusion coefficient; EA, endometrioid adenocarcinoma.

As shown in **Table 4**, the T2 and ADC values between types I and II EMC(*p* = 0.266 and *p* = 0.245, respectively),and between superficial and deep myometrial invasion (*p* = 0.793 and *p* = 0.235, respectively) did not show any statistically significant differences. The T2 and ADC values of the endometrioid adenocarcinoma (EA) were significantly lower than those of the non-EA tumors (*p* = 0.003 and *p* = 0.043, respectively). Moreover, a quantitative analysis of the EA tumors with different pathologic grades demonstrated that the T2 values were significantly lower for grade 3 (G3) EA than those for grade 1 (G1) EA or grade 2 (G2) EA (both *p* < 0.017) (**Figure 3**; **Table 4**). However, the differences in the T2 values between the G1 EA and G2 EA were not statistically significant (*p* > 0.05). Furthermore, the ADC values for the G3 EA were significantly lower than those for the G1 EA (*p* < 0.017; **Table 4**).

**Table 4:** T2 and ADC values of the clinical types, pathologic types, and grades of EMC.

Groups	T2 (ms)	ADC (×10-3mm2/s)
Tissue type		
endometrial carcinoma(n=56)	86.8 ± 10.4	1.52 ± 0.50
normal endometrium(n=17)	152.0 ± 58.0	0.73 ± 0.13
P	< 0.001	< 0.001
Clinical type		
Type I (n=36)	87.9 ± 8.7	0.71 ± 0.09
Type II (n=20)	85.0 ± 13.1	0.77 ± 0.18
P	0.266	0.245
Pathologic type		
EA (n=46)	84.9 ± 8.96	0.71 ± 0.10
Non-EA (n=10)	94.9 ± 10.5	0.84 ± 0.19
P	0.003	0.043
Tumor grade (EA)		
Grade 1 (n=19)	91.0 ± 9.1	0.76 ± 0.08
Grade 2 (n=17)	83.8 ± 3.1	0.70 ± 0.09
Grade 3 (n=10)	75.0 ± 5.6ab	0.64 ± 0.06a
P	< 0.001	0.002
Myometrial invasion		
Superficial (n=40)	87.1 ± 9.2	0.76 ± 0.14
Deep (n=16)	85.6 ± 11.9	0.71 ± 0.11
P	0.793	0.235

a *P* < 0.017 (significantly different from grade 1); ^b^*P* < 0.017 (significantly different from grade 2).

ADC, apparent diffusion coefficient; EMC, endometrial carcinoma; EA endometrioid adenocarcinoma.

### 3.4 Comparison of ROC curves

**Figure 4:**
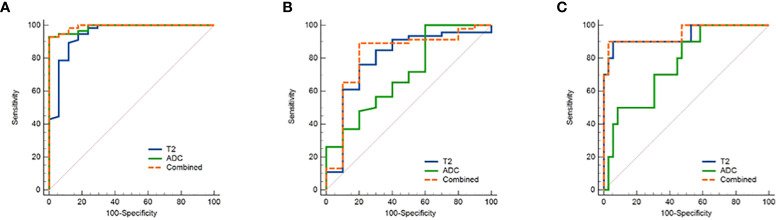
ROC curves show the diagnostic accuracy of the T2, ADC values and the combination of T2 and ADC values in distinguishing **(A)** between endometrial carcinoma and normal endometrium and **(B)** between EA and non-EA tumors and **(C)** between G3 EA and G1/G2 EA tumors. Abbreviations: ROC, receiver operating characteristic; T2, transverse relaxation time; ADC, apparent diffusion coefficient; EA, endometrioid adenocarcinoma.

**Figure 5:**
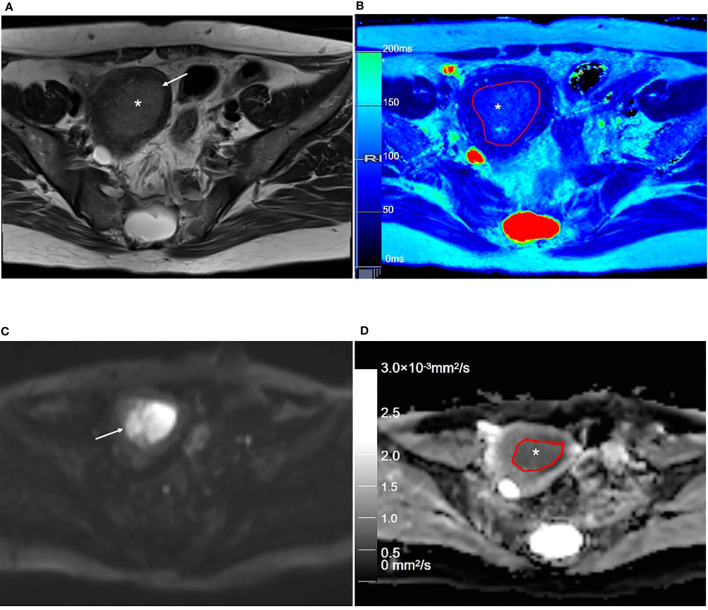
Representative T2WI and DWI and the corresponding axial T2 and ADC maps of the endometrium region of a 53-year-old patient with stage IA endometrial carcinoma. **(A)** Axial T2-weighted image shows there is an extensive, hyperintense mass lesion (*) confined to EMC in the uterine cavity. **(B)** Axial T2 map shows that the mass lesion (outlined in red, * in **B**) is slightly hyperintense. **(C)** Axial DWI (b = 1000 s/mm^2^) shows that the mass lesion (arrow) is hyperintense. **(D)** The axial ADC map shows that the mass lesion (outlined in red, * in **D**) is hypointense. T2WI, T2-weighted imaging; DWI, diffusion-weighted imaging; ADC, apparent diffusion coefficient; JZ, junctional zone.

The threshold, AUC, p value, sensitivity, and specificity of T2 and ADC values discriminating three categories (EMC/normal, EA/non-EA, and tumor grade) are listed in **Table 5**. The AUC values for the T2 and ADC values in distinguishing between the EMC and normal endometrium were 0.944 and 0.987, respectively (**Figures 4A** and **5**), with no significant difference (DeLong test: Z = 1.212; *p* = 0.226). When combined T2 and ADC values, the AUC showed a slight increase to 0.992 with no significant difference (*p* = 0.160 and 0.390, respectively). The AUC values for the T2 and ADC values in distinguishing between the EA and non-EA tumors were 0.798 and 0.705, respectively (**Figure 4B**), with no significant difference(DeLong test: Z = 0.851; *p* = 0.395). When combined T2 and ADC values, the AUC showed a slight increase to 0.817 with no significant difference (*p* = 0.663 and 0.125, respectively).The AUC values for predicting G3 EA were 0.939 for the T2 values and 0.764 for the ADC values (**Figure 4C**). According to the DeLong test, these differences were statistically significant (Z=1.972; *p* = 0.048), that is, however,slightly less significant. When combined T2 and ADC values, the AUC showed a significant increase to 0.947 compared with the ADC values (p = 0.03, **Figure 4C**).

**Table 5:** Diagnostic performance of T2 and ADC values.

Category	threshold	AUC (95%CI)	P-value	Sensitivity	Specificity
EMC vs normal					
T2 values (ms)	101.7	0.944 (0.864 - 0.984)	< 0.001	89.3%	84.2%
ADC values (×10-3mm2/s)	0.96	0.987 (0.928 - 1.000)	< 0.001	92.9%	100%
Combined	NA	0.992 (0.935 - 1.000)	< 0.001	92.9%	100%
EA vs Non-EA					
T2 values (ms)	87.8	0.798 (0.669-0.893)	< 0.001	76.1%	80.0%
ADC values (×10-3mm2/s)	0.94	0.705 (0.569-0.820)	0.03	100%	40.0%
Combined	NA	0.817 (0.691-0.908)	< 0.001	89.1%	80.0%
G3 vs G1/2					
T2 values (ms)	79.5	0.939 (0.827-0.988)	< 0.001	90.0%	94.4%
ADC values (×10-3mm2/s)	0.71	0.764 (0.616-0.876)	< 0.01	90.0%	52.8%
Combined	NA	0.947 (0.838-0.991)	< 0.001	90.0%	97.2%

ADC, apparent diffusion coefficient; AUC, area under the curve; CI, confidence interval; EA, endometrioid adenocarcinoma; NA, not applicable.

### 3.5 Correlation analysis

**Figure 6:**
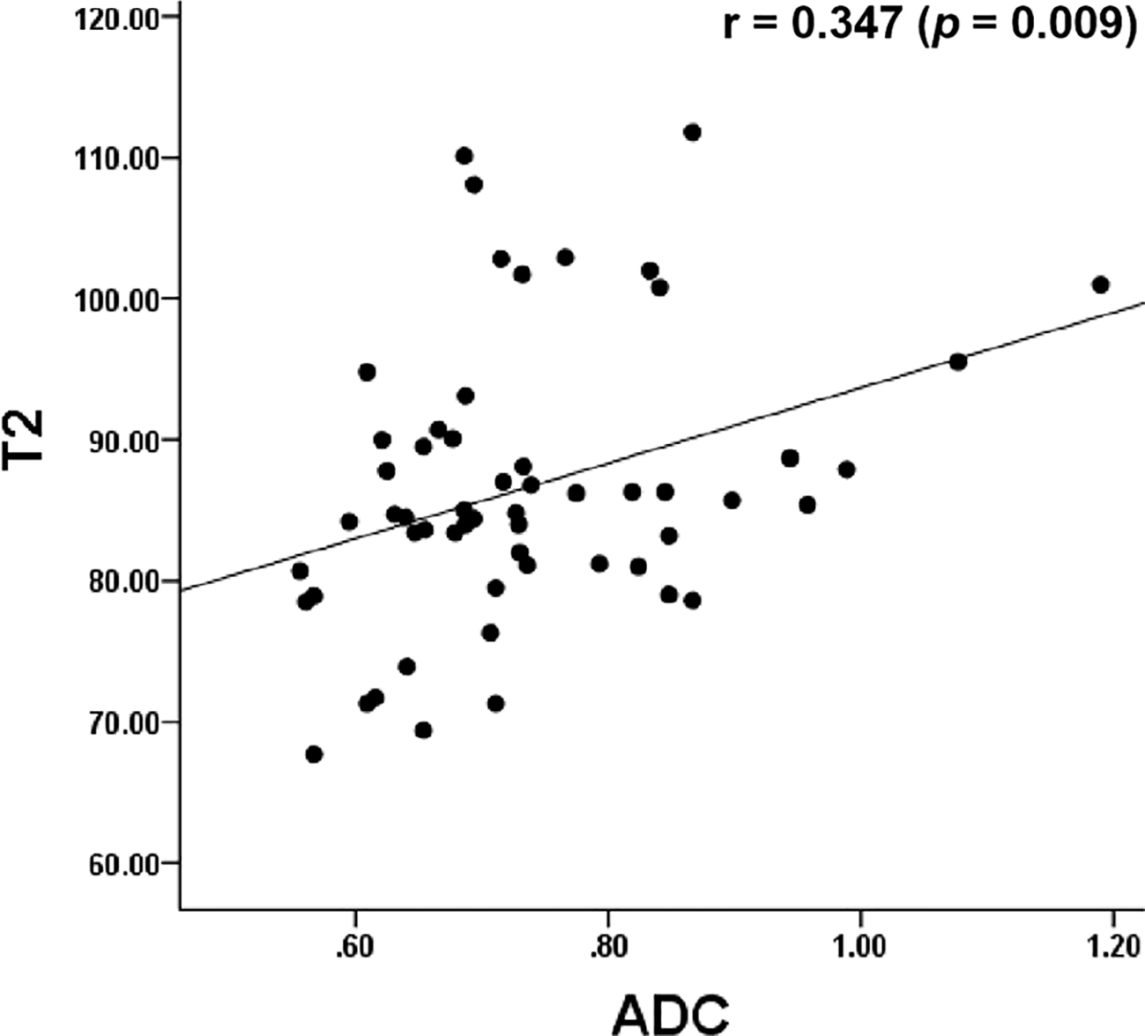
Dot graph show the correlation between T2 and ADC values. ADC, apparent diffusion coefficient.

The Pearson’s correlation analysis showed that there was a significant positive correlation between the T2 and the ADC values (r=0.347, p=0.009, **Figure 6**). The correlation of the histological grades with the T2 and ADC values showed a significant inverse correlation with r = -0.706 (P < 0.001) and r = -0.517 (P < 0.001), respectively.

## 4 Discussion

This study evaluated the feasibility of using accelerated T2 mapping for the preoperative evaluation of EMC. Quantitative T2 values accurately distinguished EMC from normal endometrial tissues, EA from non-EA, well/moderately differentiated (G1/G2) and poorly differentiated (G3) EA. The combination of T2 and ADC values resulted in a notable increase in the AUC for predicting grade 3 EA, when compared with ADC values alone. Furthermore, T2 values show a strong inverse correlation with pathologic EA grading(r=-0.706, P < 0.001).

Regarding uterine MRI, conventional pelvic MRI sequences have been used to characterize the signal intensity of normal endometrium, myometrium, and cancerous lesions by visual analysis. However, precise discernment is hindered when the signal difference between the cancerous lesion and adjacent tissue is small. T2 mapping can be used to analyze the changes in the composition of the tissue by measuring the T2 relaxation time, which is related to the water content in the tissue (16). Previous researchers have reported the value of T2 mapping in the detection and prediction of tumor histopathological features (16, 17), but few quantitative studies have focused on T2 mapping in EMC. In this study, T2 mapping can be used to visualize the morphological structure of the myometrium, especially the JZ. The JZ demonstrated a lower T2 value than the normal endometrium, outer myometrium, and EMC. The clear description of the uterine wall layers and EMC depends on the alternating differences in the T2 values of the individual layers, which may be caused by the considerable interlayer differences in cell density (5).

Our study demonstrated that quantitative T2 values could differentiate EMC from normal endometrium, which was consistent with tumors in other organs (8, 10, 16, 17). As for the ADC values, the same applied with the quantitative T2 values, a lower value is suspected to be cancer. The AUC for T2 and ADC values to differentiate EMC and normal endometriumwere 0.944 and 0.987, with no significant difference. This suggests that both parameters therefore seem to be equally suitable for the detection of EMC. Our result indicated that T2 had a weak positive correlation with the ADC values. As we know, ADC value is calculated through linear regression from DWI images taken at various b-values and is based on the restriction of water molecule diffusion within tissues. In contrast to ADC values, T2 values reflect the tissue composition, mainly free water content. Nevertheless, it has been demonstrated that ADC values as well as quantitative T2 values are linked to the cell density (18).

EMC is classified into EA, serous uterine carcinoma, undifferentiated carcinoma, uterine carcinosarcoma, and clear cell carcinoma by the 2014 World Health Organization Classification of Tumors of Female Reproductive Organs (15). Among these tumors, non-EA tumors are less prevalent than EA and show higher malignancy and poorer prognosis (15). The results of the current study showed that the T2 values of the non-EA group were significantly higher than those of the EA group. This may be related to the mixed tissue composition of the carcinosarcoma and the clear cell carcinoma, with the non-EA groups of EMC having more micronecrotic foci and cystic epithelial components than the EA groups of EMC (19, 20). Furthermore, extensive tumor micronecrosis in the serous uterine carcinoma and the undifferentiated carcinoma may contribute to the higher T2 values of the non-EA groups of EMC. This study showed that the diagnostic accuracy of the T2 and ADC values in distinguishing EA and non-EA tumors is comparable. However, we did not observe significant differences in the T2 and ADC values between types I and II EMCs, and between superficial and deep myometrial invasion, which was consistent with previous studies (6).

The pathologic grade of EMC is a major prognostic factor that affects the survival rates of patients with EMC (21). In the present study, only the pathologic grade of the EA tumors was analyzed because the number of non-EA cases was small in the study cohort. With regard to the ADC values, a significant inverse correlation with the EA differentiation grades has already been shown in current and previous studies (4, 7). Similarly, the quantitative T2 values decrease with increasing differentiation grades with r=0.706. Gu et al. (17) demonstrated that the T2 values for brain tumors with higher cell density were lower because of reduced extracellular space. Adams et al. (22) reported that the T2 values for high-grade renal clear cell carcinoma were significantly lower than those for low-grade renal carcinoma, and these findings were consistent with the results of the current study, in which the T2 values of G3 EA were lower and associated with reduced glandular structure and increased solid components. In addition, higher cell density and nuclear-to-cytoplasmic ratio in the G3 EA tumors decreased the extracellular space and reduced the water content in the tumor tissues. For prediction of poorly differentiated (G3) EA, T2 values yielded an AUC of 0.94, which means that the T2 mapping can provide valuable insights into the histological grades of EA. Combining the T2 value to ADC values improved the AUC by up to 0.947 with significant difference, when compared to the AUC obtained solely from ADC values for predicting G3 EA. These results point to the potential of T2 mapping to provide information complementary to that provided by DW imaging for tumor differentiation.

The T2 values in this study were obtained on the basis of a novel fast imaging technique (i.e., GRAPPATINI) with a parallel acquisition factor of two and an undersampling factor of five. The T2 maps were reconstructed with a 10-fold undersampled k-space, which significantly reduced the acquisition time and improved the clinical utility of T2 mapping. The broad clinical application of conventional T2 mapping is limited by long image acquisition times and potential motion artifacts because of patient movements. Previous studies have confirmed that the T2 values obtained with the GRAPPATINI technique are comparable with those obtained using the conventional multiecho spin echo sequence (9, 11, 23). Therefore, accelerated T2 mapping is a fast functional imaging technique that can yield accurate T2 values for quantifying the underlying pathophysiology in biological tissues within a shorter period.

This study has a few limitations. The sample size of the study cohort was small. Furthermore, the number of cases in the non-EA group were significantly low. Therefore, analysis of the T2 values for rare tumors such as serous uterine carcinoma, uterine carcinosarcoma, and undifferentiated carcinoma was not feasible. Also, future large-scale studies are necessary to confirm our results and characterize different subgroups of EMC.

In summary, our study demonstrated that the quantitative T2 values obtained from accelerated T2 mapping were useful for preoperative diagnosis and the pathologic classification and grading of EMC.

## Data availability statement

The original contributions presented in the study are included in the article/supplementary material. Further inquiries can be directed to the corresponding authors.

## Ethics statement

The studies involving human participants were reviewed and approved by The First Affiliated Hospital of Zhengzhou University. The patients/participants provided their written informed consent to participate in this study. Written informed consent was obtained from the individual(s) for the publication of any potentially identifiable images or data included in this article.

## Author contributions

TH, TK, JZ, FQ: Conceptualization, Methodology, Software, Validation. ZZ, JL, WW: Data curation, Writing- Original draft preparation and Editing. YZ, JC: Supervision.
